# HeLa TI cell-based assay as a new approach to screen for chemicals able to reactivate the expression of epigenetically silenced genes

**DOI:** 10.1371/journal.pone.0252504

**Published:** 2021-06-11

**Authors:** Varvara Maksimova, Natalya Shalginskikh, Olga Vlasova, Olga Usalka, Anastasia Beizer, Polina Bugaeva, Dmitry Fedorov, Olga Lizogub, Ekaterina Lesovaya, Richard Katz, Gennady Belitsky, Kirill Kirsanov, Marianna Yakubovskaya

**Affiliations:** 1 Department of Chemical Carcinogenesis, Institute of Carcinogenesis, N.N. Blokhin National Medical Research Center of Oncology, Moscow, Russia; 2 Fox Chase Cancer Center, Temple University, Philadelphia, PA, United States of America; 3 International School "Medicine of the Future", Sechenov University, Moscow, Russia; 4 Department of Translational Neurobiology, Julius-Maximilians-Universität of Würzburg, Würzburg, Germany; 5 Department of Urology, A.V. Vishnevsky National Medical Research Center of Surgery, Moscow, Russia; 6 Department of Oncology, Ryazan State Medical University, Ryazan, Russia; 7 Department of General and Medical Practice, Medical Institute, The Peoples’ Friendship University of Russia, Moscow, Russia; Barts and The London School of Medicine and Dentistry Blizard Institute, UNITED KINGDOM

## Abstract

Chemicals reactivating epigenetically silenced genes target diverse classes of enzymes, including DNMTs, HDACs, HMTs and BET protein family members. They can strongly influence the expression of genes and endogenous retroviral elements with concomitant dsRNA synthesis and massive transcription of LTRs. Chemicals reactivating gene expression may cause both beneficial effects in cancer cells and may be hazardous by promoting carcinogenesis. Among chemicals used in medicine and commerce, only a small fraction has been studied with respect to their influence on epigenetic silencing. Screening of chemicals reactivating silent genes requires adequate systems mimicking whole-genome processes. We used a HeLa TSA-inducible cell population (HeLa TI cells) obtained by retroviral infection of a *GFP*-containing vector followed by several rounds of cell sorting for screening purposes. Previously, the details of GFP epigenetic silencing in HeLa TI cells were thoroughly described. Herein, we show that the epigenetically repressed gene *GFP* is reactivated by 15 agents, including HDAC inhibitors–vorinostat, sodium butyrate, valproic acid, depsipeptide, pomiferin, and entinostat; DNMT inhibitors–decitabine, 5-azacytidine, RG108; HMT inhibitors–UNC0638, BIX01294, DZNep; a chromatin remodeler–curaxin CBL0137; and BET inhibitors–JQ-1 and JQ-35. We demonstrate that combinations of epigenetic modulators caused a significant increase in cell number with reactivated GFP compared to the individual effects of each agent. HeLa TI cells are competent to metabolize xenobiotics and possess constitutively expressed and inducible cytochrome P450 mono-oxygenases involved in xenobiotic biotransformation. Thus, HeLa TI cells may be used as an adequate test system for the extensive screening of chemicals, including those that must be metabolically activated. Studying the additional metabolic activation of xenobiotics, we surprisingly found that the rat liver S9 fraction, which has been widely used for xenobiotic activation in genotoxicity tests, reactivated epigenetically silenced genes. Applying the HeLa TI system, we show that N-nitrosodiphenylamine and N-nitrosodimethylamine reactivate epigenetically silenced genes, probably by affecting DNA methylation.

## Introduction

Epigenetic gene silencing is an important mechanism of genome regulation and includes DNA methylation, histone modification, ncRNA interference and chromatin remodeling [1]. It is involved in the processes of expression and mobility of transposable elements [2,3], genomic imprinting, dosage compensation of sex chromosomes [4], and control of gene expression at different periods of cell division and differentiation [5]. Epigenetic regulation of gene transcription also enables some mechanisms of organism adaptation to environmental changes [6,7]. Exogenous factors affecting epigenetic regulation can both induce adaptive changes and disrupt key cellular processes, leading to adverse health outcomes [8–10]. Large population biomonitoring studies have revealed adverse health effects of many widespread chemicals, in particular, nutritional components, pharmacological agents and environmental pollutants [11–13]. There is an urgent need to assess the potential epigenetic consequences of environmental exposure more extensively [8,13]. More than 90,000 chemicals are currently used in commerce, and the potential for chemical exposure to affect human health via epigenetic mechanisms has been evaluated for only a small fraction (<2%) of these chemicals [14]. The field of epigenetic toxicology is complicated by a steady stream of new compounds and growth in the annual turnover of manufactured chemicals, which has doubled during the past ten years [10,15,16]. Moreover, toxicity studies are usually performed for individual chemicals, and it is impossible to predict the hazardous effects for complex mixtures of chemicals exhibiting nonlinear dose responses [8,14]. Technologies appropriate for the visualization of molecular events could be very helpful for the elucidation of unknown or persistent effects of environmental chemicals. In this respect, there is an urgent need to develop new screening methods that are scalable to medium and high throughput, have clear end points for different epigenetic events, produce low false positive and false negative rates and are cost effective [8,17].

Various *in vivo* model systems have been developed to study the epigenetic effects of xenobiotics. The greatest contributions to the study of the epigenetic effects of environmental pollutants have been made by using *Drosophila*, *Arabidopsis*, *Daphnia*, and *Xenopus laevis* [7,18,19]. *In vivo* models include intracisternal A-particle (IAP) mouse models, in which epigenetic states are reflected by changes in coat color and tail morphology. In particular, A^vy^ mice serve as very good *in vivo* epigenetic biosensors for epigenetic alterations [20]. However, the use of these organisms is limited due to functional differences in the mouse system of epigenetic regulation compared to that of humans, and the epigenetic assay is an expensive and time-consuming. Moreover, traditional animal-based tests raise significant ethical issues [13,21]. The most convenient screening assays for epigenetically active compounds are based on in vitro model systems using immortalized cells [8,10,13]. The advantages of cell-based models include low cost and shorter experiment times. Cell-based reporter assays have been developed to reveal global methylation changes [22,23] and site-specific DNA methylation alterations [24]; however, the former are not sensitive enough to reveal different site-specific changes, and the latter cannot be applied to assessments of total effects. Based on CRISPR/Cas9/Cas12a, a new test revealing multiple end points and characterized by high-throughput screening of epigenetic modifiers has been developed [25,26]. However, these systems are limited by the number of enzymes that can be subjected to genome editing. Martinez et al. described the development of a cell-based fluorescent assay to screen epigenetic modulators with different mechanisms of action [27,28]. In this system, C127 mouse mammary adenocarcinoma cells were transfected with expression vectors to generate a *GFP*‐tagged construct driven by the CMV promoter and an aminoglycoside phosphotransferase, which confers neomycin resistance. Then, the neomycin-resistant cells testing consistently negative for *GFP* expression were selected for further use in assays for screening chemicals that reactivate *GFP* expression. Although the authors used known inhibitors of different enzymes, notably, trichostatin A (TSA), sodium butyrate and 5-azacytidine (5-azaC), as positive controls, the *GFP*-silencing mechanisms were not analyzed. To further develop the cell-based reporter approach for epigenetic modulator screening, we propose using HeLa TSA-inducible (HeLa TI) cells that harbor a silent avian sarcoma virus-based *GFP* vector [29,30]. This HeLa TI cell population has been previously obtained by multiple consecutive process to sort cells with silenced *GFP*. The HeLa TI cell population has been used to study the mechanisms of epigenetic control of retroviruses, and more than 15 various chromatin-modifying enzymes and the DAXX histone chaperone were found to participate in *GFP* silencing [31,32].

Thus, a HeLa TI-based assay is proposed to screen xenobiotics or their mixtures reactivating epigenetic silencing. One of the main goals of the present study was the demonstration of *GFP*-reactivating effects for known epigenetic modulators influencing different components of the epigenetic regulation system and of the comprehensiveness of the assay proposed. Environmental pollution represents mixtures of xenobiotics, which is why applying the HeLa TI system to reveal the combinational effects of different xenobiotics is an interesting approach. To this end, we analyzed the effects of combinations of epigenetic modulators and compared them to their individual effects.

Another goal was to examine whether HeLa TI cells harbor enzymes required for xenobiotic biotransformation. It has been previously shown that only a small group of exogenous chemicals is excreted unchanged in urine or feces without any metabolic degradation, while the majority of chemicals undergo biotransformation to active metabolites that ultimately lead to biological effects [33]. Primarily based on the work of James and Elizabeth Miller, it was established that the majority of carcinogens are chemically stable and require bioactivation to be capable of interacting with DNA [34]. Currently, the safety assessment of chemicals includes biotransformation by liver enzymes or by the S9 fraction in genotoxicity test protocols performed *in vitro* [35–38]. Many epigenetically active compounds, including the main carcinogenic biosphere pollutants TCDD [39], bisphenol A [40], benzene [41], and endosulfan-α [42], are subjected to biotransformation by cytochrome P450 monooxygenases, the main group of enzymes involved in xenobiotic metabolism. Moreover, the ultimate effects of TCDD and bisphenol A are dependent on metabolic activation patterns [39,40]. Previously, Petrowska et al. and Iwanari et al. demonstrated that HeLa cells possess active cytochrome P450 isoforms [43,44]. However, we cannot be confident that, during several rounds of directed cell selection under the influence of TSA, some changes in microsomal oxygenases has not occurred, as this compound has been shown to modify cytochrome P450 activity [45,46]. In toxicology assays, in vitro metabolic activation of xenobiotics has been performed using the rodent liver S9 fraction, consisting of cytosol and microsomes, for more than 30 years [37,47]. Thus, the next goal of our work was to investigate the influence of S9 on the reactivation of epigenetically silenced genes. The final goal of our study was the application of the HeLa TI system for testing a number of procarcinogens from the nitrosamine group.

## Materials and methods

### Chemicals and reagents

#### Epigenetic modulators

***Histone deacetylase inhibitors (HDACis)*:** trichostatin A, (TSA, CAS 58880-19-6, (1)), vorinostat (CAS 149647-78-9, (1)), sodium butyrate (CAS 156-54-7, (1)), valproic acid (VPA, CAS 99-66-1, (1)), depsipeptide (PubChem 5352062, (1)), pomiferin (CAS 572-03-2 (1)), entinostat (CAS 209783-80-2, (1)); ***histone methyltransferase inhibitors (HMTis)***: 3-deazaneplanocin A (DZNep, CAS 1020,52-95-9(1)), tazemetostat (TAZ, CAS 1403254-99-8, (2)), A-196 (CAS 1982372-88-2, (2)), UNC0638 (CAS 1255580-76-7, (2)), BIX01294 (CAS 1392399-03-9, (1)); ***lysine demethylase inhibitor (KDMi)*** GSK2879552 (CAS 1401966-69-5, (2)); ***DNA-methyltransferase inhibitors (DNMTis)*:** 5-azacytidine (5-azaC, CAS 320-67-2, (1)), decitabine (DAC, CAS 2353-33-5 (1)), RG108 (CAS 48208-26-0, (1)); ***Bromodomains and Extra-Terminal motif inhibitors (BETis)*:** JQ-35 (CAS 1349719-98-7, (2)), JQ-1 (CAS, 1268524-70-4, (1)); ***chromatin remodeler*** curaxin CBL0137 (CBL0137, CAS, 1197996-80-7, (2)). All of the above compounds were obtained from Sigma Aldrich (Merck) (1) and Selleckchem (2). All agents were dissolved in dimethyl sulfoxide (DMSO) with the exception of sodium butyrate and valproic acid, which were dissolved in water.

#### Carcinogens

Benzo[a]pyrene (BaP, CAS 50-32-8), 1,12-benzoperylene (CAS 191-24-2), 3-methylcholanthrene (3-MC, CAS 56-49-5), aflatoxin B1 (CAS 1162-65-8), o-aminoazotoluene (CAS 97-56-3), rifampicin (CAS 13292-46-1), 4-nitroquinoline-1-oxide (4-NQO, CAS 56-57-5), cisplatin (CAS 15663-27-1) were purchased from Sigma Aldrich (Merck). Isoniazid (CAS 54-85-3), cyclophosphamidum (CAS 50-18-0), phenobarbital (CAS 50-06-6) were obtained from Baxter Oncology. Nitrosamines N-nitrosodiallylamine (NDAA, CAS 16338-97-9), N-nitrosodibutylamine (NDBA, CAS 924-16-3), N-nitrosodimethylamine (NDMA, CAS 62-75-9), N-nitrosodipropylamine (NDPA, CAS 621-64-7), N-nitrosodiphenylamine (NDPhA, CAS 86-30-6), N-nitrosodiethylamine (NDEA, CAS 55-18-5) were purchased from Sigma Aldrich (Merck).

#### Other

Phosphate-buffered saline (PBS, P4417) was purchased from Sigma Aldrich (Merck). DMSO (Cat Ф135) was purchased from Paneco. Low melting agarose (LMA, Cat 4250-500-02) and lysis solution (Cat 4250-010-01) for Comet assay were purchased from Trevigen. S9 fraction was obtained from laboratory of mechanisms of chemical carcinogenesis (Moscow, Russia). NADP was purchased from Applichem (Cat A1394), glucose-6-phosphate (G6P) was purchased from Reanal Laboratory Chemicals, KCl was purchased from ChemMed. GeneJET RNA Purification Kit (K0732) was purchased from Thermo Scientific. Eppendorf Cell Culture Plates (96-, 24-, 12-, 6-well) for adherent cells (EP0030730011, EP0030722019, EP0030721012, EP0030720016), Eppendorf Cell Culture Flasks T-25 and T-75 (EP0030710126, EP0030711017) were purchased from Sigma Aldrich (Merck).

### Cell cultures

HeLa TI cells were obtained from Fox Chase Cancer Center (Philadelphia, USA). CaSki cells were obtained from the Laboratory of Molecular Biology of Viruses of N.N. Blokhin NMRCO. The cells were maintained in plastic culture flasks under standard conditions (37°C, 5% CO_2_). They were transferred by washing with Versene solution and treated with buffered trypsin-EDTA 0.25% (Cat Р080п, П036п) from Paneco at a 1:5 ratio v/v every 3–4 days. The cells were maintained in Dulbecco’s modified Eagle’s medium (DMEM, Cat 12491–015) supplemented with 10% fetal bovine serum (FBS, Cat 26140079), 50 u/ml penicillin-streptomycin (Cat А065), and 200 μM L-glutamine (Cat F032), obtained from Paneco.

### Evaluation of compounds cytotoxicity

The cytotoxicity of epigenetic modulators and other compounds used in the study was measured using MTT tests [48]. The cells were seeded at 5×10^3^ cells per well in 96-well flat-bottom plates and incubated overnight. Next, serial dilutions of the preparations were added in triplicate sets and incubated for 72 hours under standard conditions. Then, the cells were treated with 3-[4,5-dimethylthiazol-2-yl]-2,5-diphenyltetrazolium bromide (MTT, D298931, Dia-M). After four hours of exposure to MTT, the medium was removed, and 100 μl of DMSO was added. The optical density of the solution was measured at 540 nm using a Multiskan Sky microplate spectrophotometer (Thermo Scientific). The cytotoxicity index was determined using 0.1% DMSO as a negative control.

### Assessment of the effects of epigenetic modulators on *GFP* reactivation in HeLa TI cells

#### Dose-dependent effects of TSA on GFP reactivation

Cells were seeded in 24-well plates at 2×10^4^ cells per well and treated with TSA (0.5 μM, 0.25 μM, 0.12 μM, 0.06 μM, or 0.03 μM) 24 hours later. After 24 hours of incubation, the medium in the plates was replaced with fresh medium, and the cells were incubated for another 48 hours. Next, the cells were detached from the culture plates using a 0.25% trypsin-EDTA solution (Paneco), washed with PBS and analyzed with flow cytometry. To maintain high cell viability, we used a PBS solution with 2% fetal bovine serum as a buffer to store the cell suspension. The maximum concentration of DMSO for dissolving TSA in the medium was 0.1%. Hereafter, this protocol is referred to as the standard protocol.

#### Time-dependent effects of epigenetic modulators on GFP reactivation

Cells were seeded in 24-well plates at 6×10^3^ cells per well and treated with nontoxic concentrations of analyzed drugs 120h, 96h, 72h, 48h, 24h, 12h, 6h, and 3h before flow cytometry. The medium in the plates was replaced with fresh one after 24h incubation with compound (for 120h, 96h, 72h, 48h experiments). Agents and concentrations: TSA (0.25 μM), 5-azacytidine (4 μM), A-196 (8 μM), UNC0638 (8 μM), tazemetostat (8 μM); GSK2879552 (1 μM); JQ-35 (8 μM), CBL0137 (0.2 μM). All of the compounds were solved in DMSO. Maximum concentration of DMSO in media was 0.1% for TSA, 5-azaC and CBL0137, while for other it was 0.4%.

#### Effects of epigenetic modulators on GFP reactivation

The cells were seeded in 24-well plates, treated and analyzed according to the standard protocol. Agents and concentrations: HDACis: TSA (0.25 μM), sodium butyrate (5 mM), depsipeptide (5 μM), entinostat (5 μM), pomiferin (5 μM), vorinostat (5 μM), VPA (5 μM); DNMTis: 5-azaC (4 μM), DAC (5 μM), RG108 (5 μM); HMTis: A-196 (8 μM), BIX01294 (5 μM), UNC0638 (4 μM), DZNep (5 μM), tazemetostat (8 μM); KDMi: GSK2879552 (1 μM); BETis: JQ-35 (8 μM), JQ-1 (5 μM), CBL0137 (0.2 μM). Maximum concentration of DMSO in media was 0.1% for TSA, depsipeptide, entinostat, pomiferin, vorinostat, DAC, RG108, BIX01294 and DZNep. For A-196, UNC0638, tazemetostat; GSK2879552, JQ-35 and JQ-1 maximum concentration of DMSO was 0.4%. For 5-azaC and CBL0137 maximum concentration of DMSO in media was 0.001%. Sodium butyrate and valproic acid were dissolved in water.

#### Effects of combinations of epigenetic modulators on GFP reactivation

Cells were seeded in 24-well plates at 2×10^4^ cells per well and treated with nontoxic concentrations (IC10) of combinations of agents from various epigenetic modulator groups 24 hours later. Then, we followed the standard protocol described above using the following agents at the indicated concentrations: HDACi TSA (0.2 μM, 0.12 μM and 0.06 μM); DNMTi 5-azacytidine (4 μM and 2 μM); HMTi UNC0638 (4 μM and 2 μM) and tazemetostat (24h treatment, 4 μM and 2 μM); BETi: JQ-35 (24h treatment, 8 μM and 4 μM), JQ-1 (5 μM and 2.5 μM); chromatin remodeler CBL0137 (0.2 μM and 0.1 μM). We assessed the combination effects of the analyzed agents with epigenetic modulators in the most common groups (HDAC inhibitors and DNMT inhibitors), as well as the chromatin remodeler curaxin CBL0137 due to its integral epigenetic effect. The maximum concentration of DMSO in the culture medium was 0.001% to dissolve TSA, 5-azaC and CBL0137. For A-196, UNC0638, tazemetostat, GSK2879552, JQ-35 and JQ-1, the maximum concentration of DMSO was 0.4%. Concentrations for the combination analysis were selected to ensure that under the combined action of the agents cell viability did not decrease to less than 85–90%.

### Assessment of the effects of procarcinogens on *GFP* reactivation in HeLa TI cells

Cells were seeded in 24-well plates, treated and analyzed according to the standard protocol. Compounds and concentrations: NDAA (40 μM), NDBA (40 μM), NDMA (40 μM), NDPA (40 μM), NDPhA (20 nM), NDEA (40 μM). Nitrosoamines were supplied in methanol and then diluted with water. Maximum concentration of solvent in media was 0.1%.

### Flow cytometry

Analysis of the level of reactivation of the *GFP* gene was carried out using a FACSCanto II flow cytometer and BD FascDiva Software (Becton Dickinson). The fluorescence of the GFP-positive cells was recorded in fluorescent green dye fluorescein isothiocyanate (FITC) mode. HeLa TI cells untreated and treated with a solvent at the same concentration as the analyzed agent were used as a negative controls.

### Assessment of the sensitivity of HeLa TI and CaSki cells to demethylating agents

HeLa TI and CaSki cells were seeded in 6-well plates (1.5x10^5^ cells and 2x10^5^ cells per well) and incubated with demethylation agents (5-azacytidine and decitabine, 1 μM for both). After every 24 hours of incubation, one-half of the culture medium was replaced with fresh medium, and the agents were added to the original concentration. Genomic DNA was extracted from the cells using a GeneJET genomic DNA purification kit (K0721, Thermo Scientific). The level of global DNA methylation after compound treatment was determined using a MethylFlash global DNA methylation (5-mC) ELISA easy kit (P-1030-96, EpiGentek) according to the manufacturer’s protocol. The maximum concentration of DMSO in the medium was 0.01% for dissolving 5-azacytidine and decitabine.

### Assessment of the effect of N-nitrosamines that caused GFP gene reactivation on DNA methylation

CaSki cells were seeded in 6-well plates (2x10^5^ cells per well) and incubated with 5-azacytidine (1 μM) and N-nitrosamines that caused GFP gene reactivation (NDPhA (1 μM) and NDMA (5 μM)) for 72 hours. Treatment, incubation, DNA isolation and methylation analysis by ELISA were performed as previously described.

### Assessment of the activity of biotransformation enzymes in HeLa TI cells

Here, we adapted Shpol’skii spectrometry [49] to analyze the residual amount of benzo[a]pyrene in the culture medium. For analysis, the culture medium was taken 24 and 48 hours after the treatment, and then, the substance was extracted by incubating one volume of medium with two equal volumes of n-octane for an hour on a shaker (80 rpm). A hydrocarbon fraction was selected. Then, the hydrocarbon fraction, standard solution and n-octane were mixed at a ratio of 1:1:1 v/v, and spectrophotometry was carried out at the temperature of liquid nitrogen (77 K). As a standard, a solution of 1,12-benzoperylenes in n-octane with a mass concentration of 100 μg/ml was used. The luminescence of benzo[a]pyrene and 1.12-benzapyrilene was excited at a wavelength of 367 nm. The spectrum of the solution was recorded in the wavelength region 401–409 nm. The content of benzo[a]pyrene in the sample was quantified from the ratio of the peak heights of the maxima of the characteristic benzo[a]pyrene and 1.12-benzoperylene lines at 403 nm and 406.3 nm, respectively.

### Comet assay

Cells were seeded in 12-well plates at 4×10^4^ cells per well and treated with genotoxic carcinogens 24 hours later. The following compounds were used in the experiment: benzo[a]pyrene (10 μM), 4-nitroquinoline-1-oxide (26 nM), 3-methylcholanthrene (30 μM) and cisplatin (0.8 mM). After 48 hours of incubation, the cells (3×10^5^ cells/ml) were removed from the substrate, washed with PBS and resuspended in 0.5% LMA at a ratio of 1:9 v/v. Then, 100 μl of this solution was applied to the surface CometSlide (Cell Biolabs) and allowed to set at 4°C for 15 min. Slides were dipped into cell lysis buffer for 1 h at 4°C. Next, the slides were washed with deionized water to remove detergent and salt from the microgels, and then, the slides were immersed in alkaline unwinding solution (200 mM NaOH and 1 mM EDTA, pH > 13) for 30 min at 4°C. The slides were then washed with TBE and placed in a horizontal electrophoresis unit containing cooled TBE, and electrophoresis was carried out for 25 min at an average of 1 V/cm. The slides were then washed with deionized water and fixed in ice-cold 70% ethanol for 5 min. Next, the slides were air-dried at 4°C, and each slide was stained with 100 μl of the DNA fluorescent dye SYBR Gold in TE buffer (1:10.000) at 4°C for 15 min in the dark. The slides were observed with a Zeiss AxioVert 200 fluorescence microscope with an EBQ isolated lamp at 5x magnification. At least 100 cells were obtained for each sample and analyzed using CometScore Tutorial software to measure DNA damage.

### Quantitative reverse transcriptase-polymerase chain reaction (RT-qPCR)

Intact cells were used to analyze the constitutive mRNA level. To analyze CYP isoform induction, cells were seeded in 6-well plates at 2.5×10^5^ cells per well and treated with inductors 24 hours later. The following agents were used as inducers: 3-methylcholanthrene (2 μM), aflatoxin B1 (0.2 μM), 3’,3-diaminobenzidine (80 μM), phenobarbital (1 mM), cyclophosphamide (0.15 mM), rifampicin (0.35 mM), and isoniazid (100 mM). 3-Methylcholanthrene, aflatoxin B1 and 3’,3-diaminobenzidine were dissolved in DMSO, the maximum concentration of which was less than 0.001% in the medium. Phenobarbital, cyclophosphamide, rifampicin and isoniazid were dissolved in PBS. After 48 hours of incubation with these substances, the cells were washed with PBS, and mRNA was isolated with a GeneJET RNA purification kit according to the manufacturer’s instructions. For analysis of mRNAs, total RNA was reverse transcribed to form complementary DNA with RT reagents from Syntol according to the manufacturer’s instructions. Levels of CYP1A1, CYP1A2, CYP1B1, CYP2A6, CYP2B6, CYP2C9, CYP2C19, CYP2E1, and CYP3A5 mRNAs were analyzed by quantitative PCR in real-time with detection using EvaGreen intercalating dye from Biotium. Each PCR was based on 5 ng of DNA, 1x PCR Buffer, 0.3 mM dNTPs, 3 mM MgCl_2_, 0.2 U Syn Taq DNA polymerase, and 0.2 μM forward and reverse primers in a 25-μl reaction volume. RT-qPCR was carried out with a CFX96 Touch™ Real-Time PCR detection system from Bio-Rad Laboratories. Primer sequences are provided in the Supplementary materials. Syntol provided the reagents and primers.

### Statistical analysis

To evaluate the efficiency and reproducibility of the screening protocol, the Z’-factor was calculated [31,32]; it was measured in three independent experiments using the primary reporter cell population treated with 0.25 μM TSA (positive control) and 0.1% DMSO (negative control) 48 hours after the 24-hour cell treatment. The Z’-factor was calculated as (1–3×(SD_TSA_+SD_DMSO_)/(M_TSA_−M_DMSO_)), with SD_TSA_ and SD_DMSO_ being the standard deviation and M_TSA_ and M_DMSO_ being the mean of the relative number of cells with reactivated *GFP*. The average Z’-factor was 0.802, which confirms the efficiency and reproducibility of the screening protocol.

We compared data of the treatments and controls using one-way analysis of variance (ANOVA) and Dunnett’s post hoc test. For statistical analysis of the effects of epigenetic modulator mixtures, we used one-way ANOVA and Tukey’s multiple-comparison posttest. We compared time-dependent effects of epigenetic modulators on GFP reactivation using two-way ANOVA with Dunnett’s post hoc test. Differences between groups were considered to be significant at a p value of <0.05. Statistical analyses were performed with GraphPad Prism 8.3.0.

## Results

### Assessment of the effect of epigenetic modulators on *GFP* reactivation in HeLa TI cells

#### Dose- and time-dependent effects of TSA on GFP reactivation

**Fig 1:**
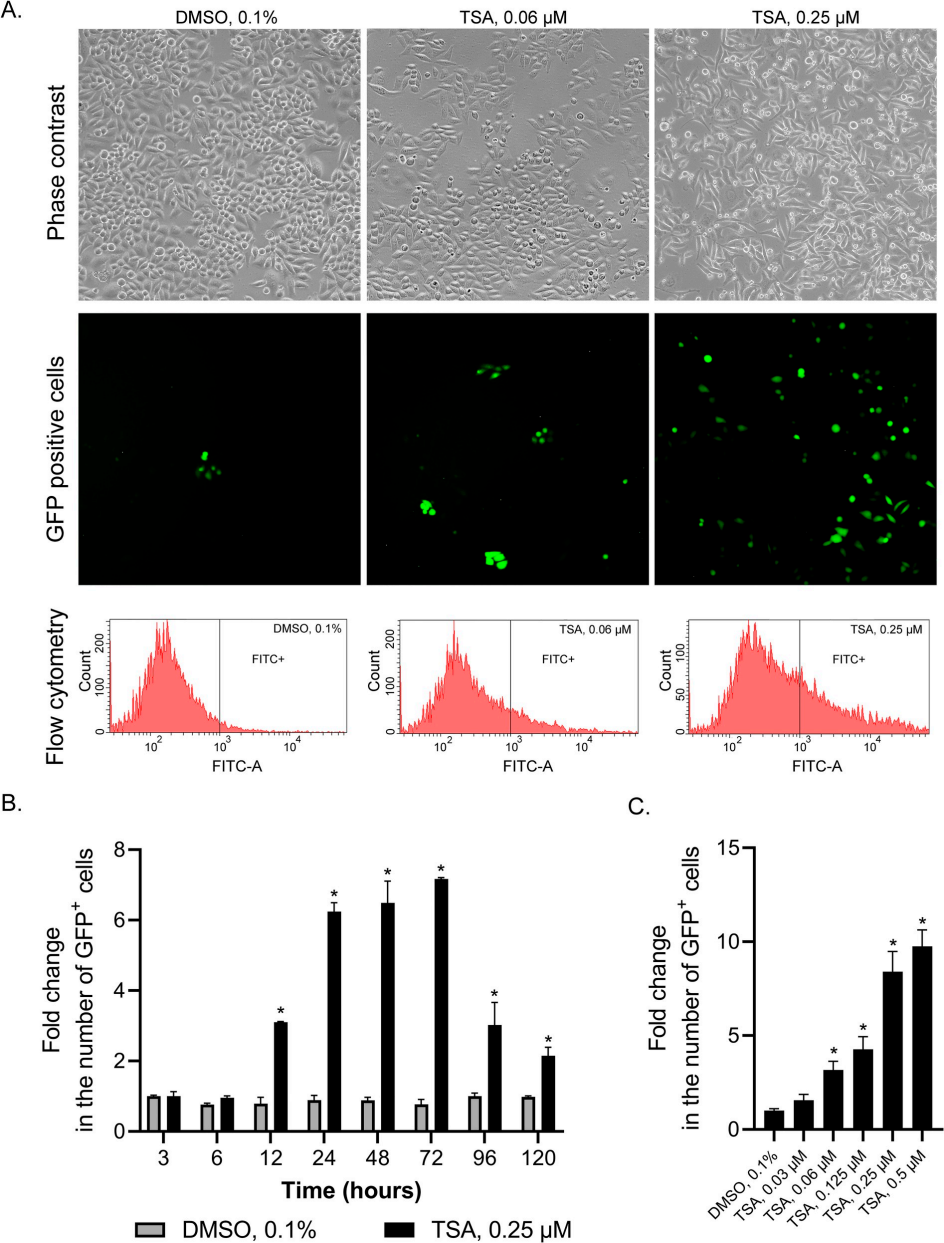
Reactivation of *GFP* expression in HeLa TI cells by TSA. A. *GFP* expression in HeLa TI cells depending on the treatment. Cells treated with TSA (0.25 μM and 0.06 μM) and DMSO (0.1%) were analyzed with phase-contrast and fluorescent microscopy and flow cytometry. B. Time-response effect of TSA; FACS. C. Dose-response effect of TSA; FACS. The values are expressed as the means ± SD; “*” indicates the level of significance to vehicle (p <0.05).

The responsiveness of the assay was demonstrated by monitoring and recording the performance against negative and positive controls, showing the concentration-response relationship for the latter [50]. In the proposed assay, TSA, a histone deacetylase inhibitor, was added to the culture medium of HeLa TI cells at different concentrations, while 0.1% DMSO was used as the vehicle control (Fig 1A). Using flow cytometry, we assessed the *GFP*-reactivating effect of TSA in the concentration range of 30–500 nM at various time points (Fig 1B). The treatment of HeLa TI cells with TSA was followed by an increase in the fraction of GFP-positive cells in a dose-dependent manner. We also analyzed the reactivation of the *GFP* gene upon exposure to TSA for a time ranging from 3 to 120 hours. A significant increase in the *GFP* gene of positive cells occurred at 24–72 hours. Thus, the dynamics of *GFP* reactivation were time-dependent. For all concentrations used, cell survival did not decrease to less than 90%.

#### Effect of different durations of treatment with epigenetic modulators on GFP expression in HeLa TI cells

To understand the time-dependence limits of HeLa TI cell application as a test system, we analyzed the reactivation of the *GFP* gene after treatment with agents from various groups of epigenetic modulators in the time range from 3 to 120 hours (S1 Fig). The majority of compounds reactivated *GFP* gene expression 72 hours after cell treatment. However, for several compounds, the maximum activity was observed after 24 hours of treatment. The data obtained were concordant with the results of the TSA time-dependence experiment. Thus, it is not recommended that the HeLa TI test system to analyze the effects of xenobiotics on epigenetic silencing for exposure times shorter than 24 hours or longer than 72 hours.

#### Effects of epigenetic modulators on GFP expression in HeLa TI cells

**Fig 2:**
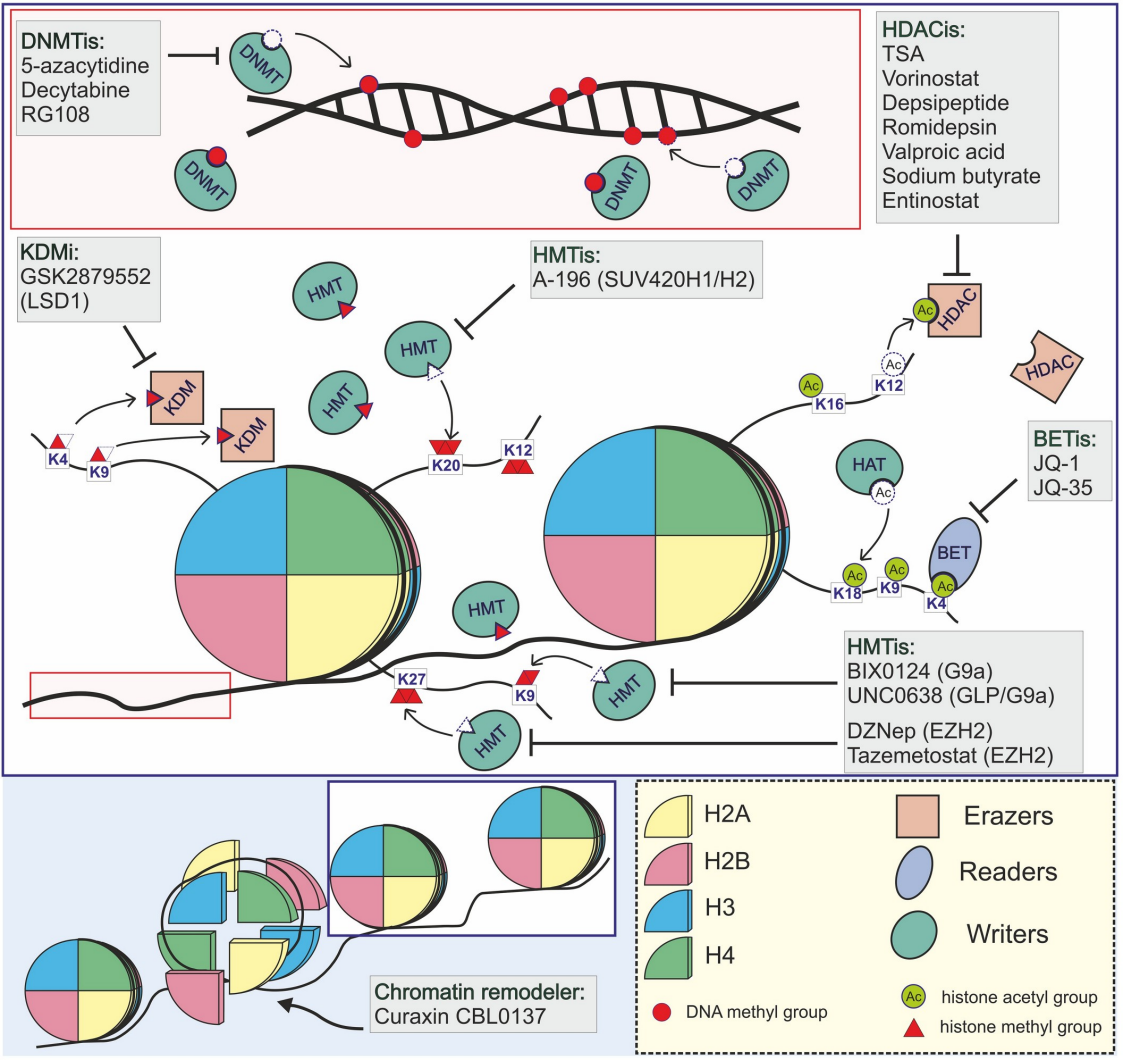
Epigenetic modulators used in the study. HDACis–histone deacetylase inhibitors, DNMTis–DNA-methyltransferase inhibitors, HMTis–histone methyltransferase inhibitors, BETis–bromodomains and extraterminal motif inhibitors, and KDMi–a lysine demethylase inhibitor.

To confirm the suitability of the HeLa TI cell population as a test system for the screening of compounds with various mechanisms of epigenetically silenced gene reactivation, we analyzed *GFP* expression under the influence of well-known epigenetic active agents. We chose agents with different epigenetic modulating activities for these experiments: histone deacetylase inhibitors (HDACis), DNA-methyltransferase inhibitors (DNMTis), histone methyltransferase inhibitors (HMTis), bromodomains and extraterminal motif inhibitors (BETis), lysine demethylase inhibitors (KDMis) and chromatin remodelers (Fig 2). The group of HDAC inhibitors included agents targeting both individual enzymes of the HDAC class and with pan-activity against vorinostat, valproic acid, sodium butyrate, depsipeptide, pomiferin, and entinostat. The group of DNMT inhibitors included the following compounds: 5-azacytidine, decitabine and RG108. The group of inhibitors of histone methyltransferases included agents BIX01294, UNC0638, DZNep, tazemetostat and A-196, which inhibit enzymes G9a, GLP, EZH2 and SUV420H1/H2, which are critical for the modifications H3K9me2, H3K27me3 and H4K20me3, which are associated with heterochromatin formation. The list of investigated agents also included the inhibitor GSK2879552 of lysine demethylase 1, which is critical for the “erasing” mono- and dimethylated lysine residues from histone H3. Another group of compounds consisted of agents that inhibit the activity of epigenetic “readers”–the bromodomain and extra-terminal domain (BET) family–JQ-1 and JQ-35. Another agent used for analysis with this test system was curaxin CBL0137, an inhibitor of the histone chaperone that facilitates chromatin transcription (FACT). This compound causes disruption of chromatin organization via nucleosome unfolding.

**Fig 3:**
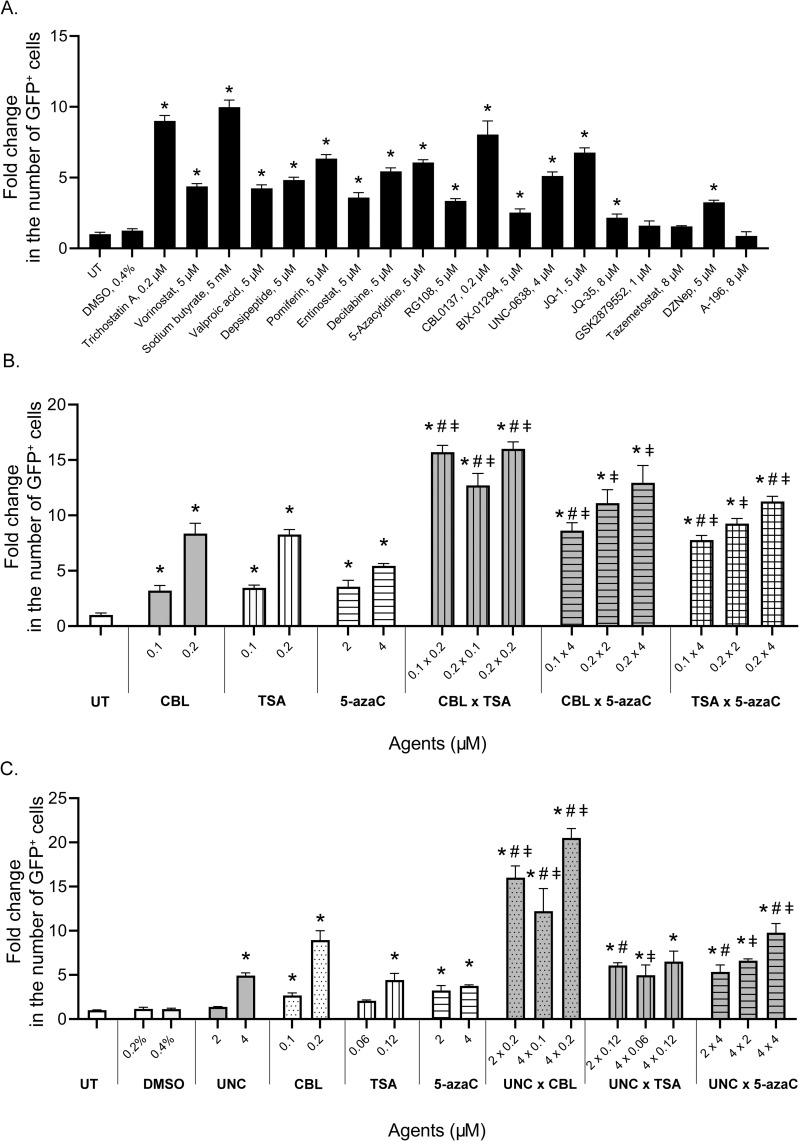
Reactivation of silenced *GFP* expression in HeLa TI cells. A. After treatment with epigenetic modulators. B. After treatment with combinations of CBL0137, TSA and 5-azaC. C. After treatment with combinations of UNC with CBL0137, TSA and 5-azaC. GFP-positive cells were counted with flow cytometry. Data normalized to vehicle. The values are expressed as the means ± SD; “*” indicates the level of significance to vehicle (p <0.05); “#” indicates the level of significance to 1^st^ agent (p <0.05) and “ǂ” indicates the level of significance to 2^nd^ agent (p <0.05).

The experimental results presented in Fig 3A demonstrate that significant reactivation of *GFP* gene expression in the HeLa TI test system occurred after 72 hours (24 hour incubation with agents and 48 hours in fresh medium) of cell exposure to all inhibitors of histone deacetylases and DNA-methyltransferases; the chromatin remodeler CBL0137; HMT inhibitors DZNep and UNC0638; and the BET inhibitor JQ-1. It was also noted that after 24 hours of incubation with JQ-35, there was a maximum increase in the number of GFP-positive cells.

#### Effects of combinations of epigenetic modulators on GFP reactivation

To analyze the ability of the proposed test system to assess the influence of xenobiotic mixtures, we studied the effects of combinations of various epigenetic modulators on the reactivation of the *GFP* gene. For the study, we selected agents that caused reactivation of the epigenetically repressed *GFP* gene during screening: HDACi TSA, DNMTi 5-azaC, BETis JQ-35 and JQ-1, HMTi UNC0638, and chromatin remodeler CBL0137.

Analysis of the combined action of TSA, 5-azaC, and CBL0137, TSA and 5-azaC, TSA and CBL0137, 5-azaC and CBL0137 showed the strongest increase in the number of GFP-positive cells compared to the individual action of the respective agents (Fig 3B).

Analysis of the combined action of UNC0638 with TSA or CBL0137 or 5-azaC showed that the combined action of these agents promoted a high level of *GFP* reactivation, especially under the action of CBL0137 (Fig 3C).

Agents A-196 and GSK2879552, which did not cause reactivation of the *GFP* gene when tested alone, did not lead to any significant level change in the number of GFP-positive cells when applied in combination with active agents, compared to the effect of each active agent alone (data not shown).

Notably, the dose-dependent nature of the reactivation of the *GFP* gene in response to treatment with compounds was shown for all agents active in the test system. The data indicate that the HeLa TI test system is a suitable tool for analyzing the combinational effect of epigenetically active xenobiotics.

**Fig 4:**
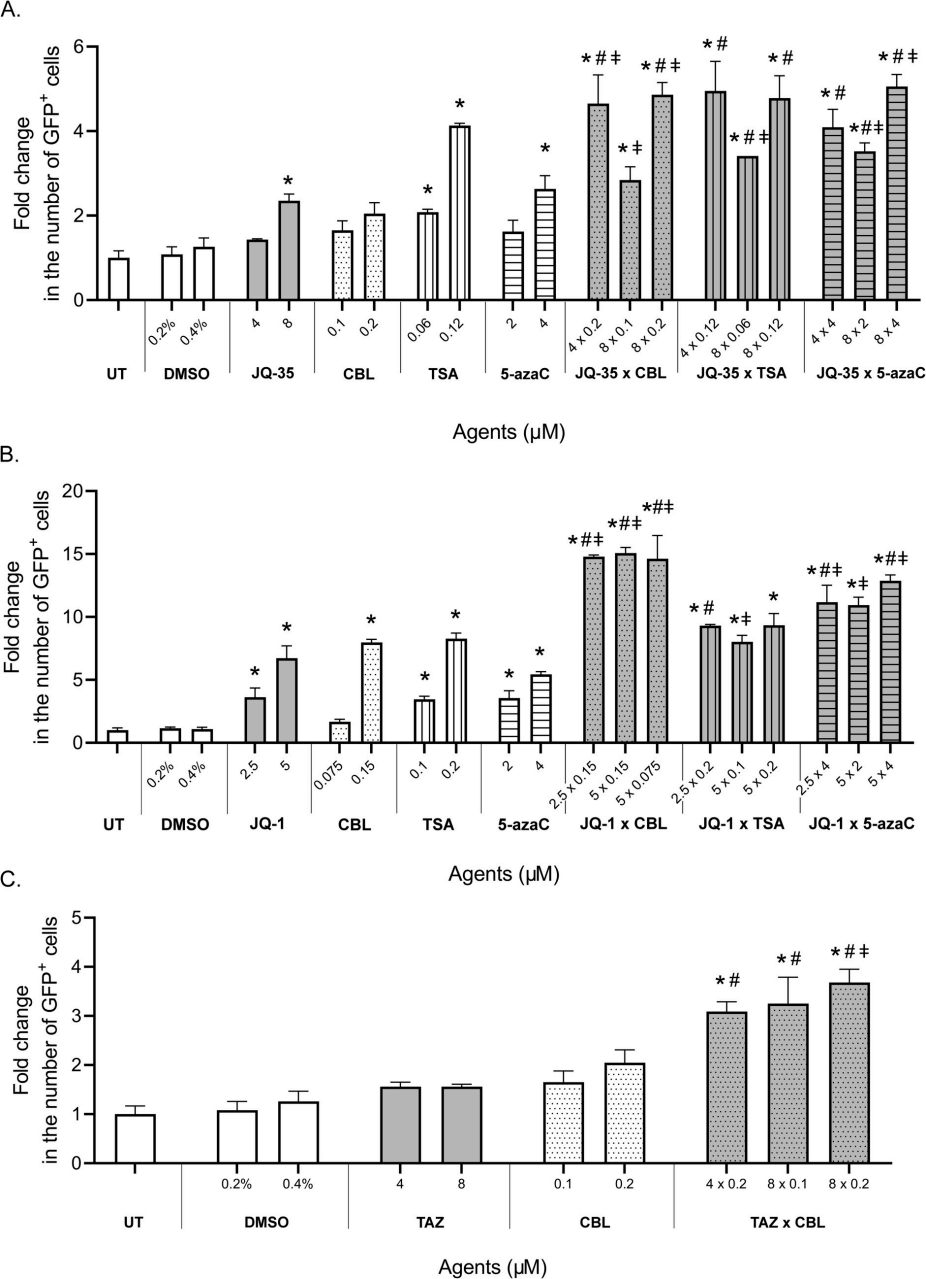
Reactivation of silenced *GFP* in HeLa TI cells by combinations of epigenetic modulators. A. JQ-35 with CBL0137, TSA and 5-azaC. B. JQ-1 with CBL0137, TSA and 5-azaC. C. Tazemetostat with CBL0137, TSA and 5-azaC. GFP-positive cells were counted with flow cytometry. Data normalized to vehicle. The values are expressed as the means ± SD; “*” indicates the level of significance to vehicle (p <0.05); “#” indicates the level of significance to 1^st^ agent (p <0.05) and “ǂ” indicates the level of significance to 2^nd^ agent (p <0.05).

After the treatment of cells with JQ-35 in combination with CBL0137 or 5-azaC, an increase in the number of GFP-positive cells was found compared to that induced by the activity of individual agents, which indicated an increase in the mutual effects of these compounds. Notably, the maximum effect of these combinations was achieved in the presence of a higher concentration of CBL0137 or 5-azaC; therefore, it can be concluded that these compounds make the main contribution to the reactivation of the *GFP* gene. When the cells were treated with JQ-35 in combination with TSA, a significant increase in GFP reactivation was observed only for the combination with a high concentration of JQ-35 and a low concentration of TSA, which may indicate a possible overlap of the effects of these agents (Fig 4A).

Analysis of the combination action of JQ-1 with TSA or 5-azaC or CBL0137 showed results similar to those observed with the JQ-35 combinations. The combined action of CBL0137 and 5-azaC caused an increase in the number of GFP-positive cells in all treatment options, while TSA combined with JQ-1 showed an increase only at the maximum concentration of JQ-1 and the minimum concentration of TSA (Fig 4B). The results obtained for JQ-1 and JQ-35 suggest that these agents have broad effects on chromatin remodeling that extend beyond their properties as inhibitors of bromodomains.

We also analyzed the effect of the drug tazemetostat, which did not cause an increase in *GFP* reactivation when tested alone, in combination with TSA, CBL0137 or 5-azaC. The experimental results showed that only when tazemetostat was combined with curaxin CBL0137 the number of GFP-positive cells increased. This effect can be explained by the remodeling activity of CBL0137, which, due to the relaxation of chromatin, could have increased the access of the tazemetostat to the target enzyme (Fig 4C).

### The system of xenobiotic biotransformation in HeLa TI cells

To assess the activity of biotransformation enzymes in HeLa TI cells, we used three alternative approaches. Overall, the ability of HeLa TI cells to metabolize procarcinogens was estimated by the decrease of the unmodified compound in the HeLa TI culture medium and by revealing the genotoxic effect of the procarcinogens on HeLa TI cells through a comet assay. Finally, we analyzed the constitutive and induced levels of cytochrome P450 isoforms, which are extremely important in xenobiotic metabolism.

### Biotransformation of procarcinogens by HeLa TI cells

Fluorescence spectroscopy using n-paraffin solutions at low temperature (known as Shpol’skii spectroscopy) was developed for the determination of parent PAHs and their derivatives, and then, it was successfully applied in the fields of environmental chemistry, toxicology and organic geochemistry [49]. We used Shpol’skii spectroscopy to determine changes in the benzo[a]pyrene concentration in the culture medium of HeLa TI cells. Benzo[a]pyrene is a well-known and widespread procarcinogen that is bioactivated by the cytochrome P450 system with the subsequent formation of carcinogenic metabolites. The main enzymes metabolizing benzopyrene are cytochromes CYP1A1 and CYP1B1. These cytochromes take part in the bioactivation of polycyclic aromatic hydrocarbons and other carcinogenic compounds [51].

**Fig 5:**
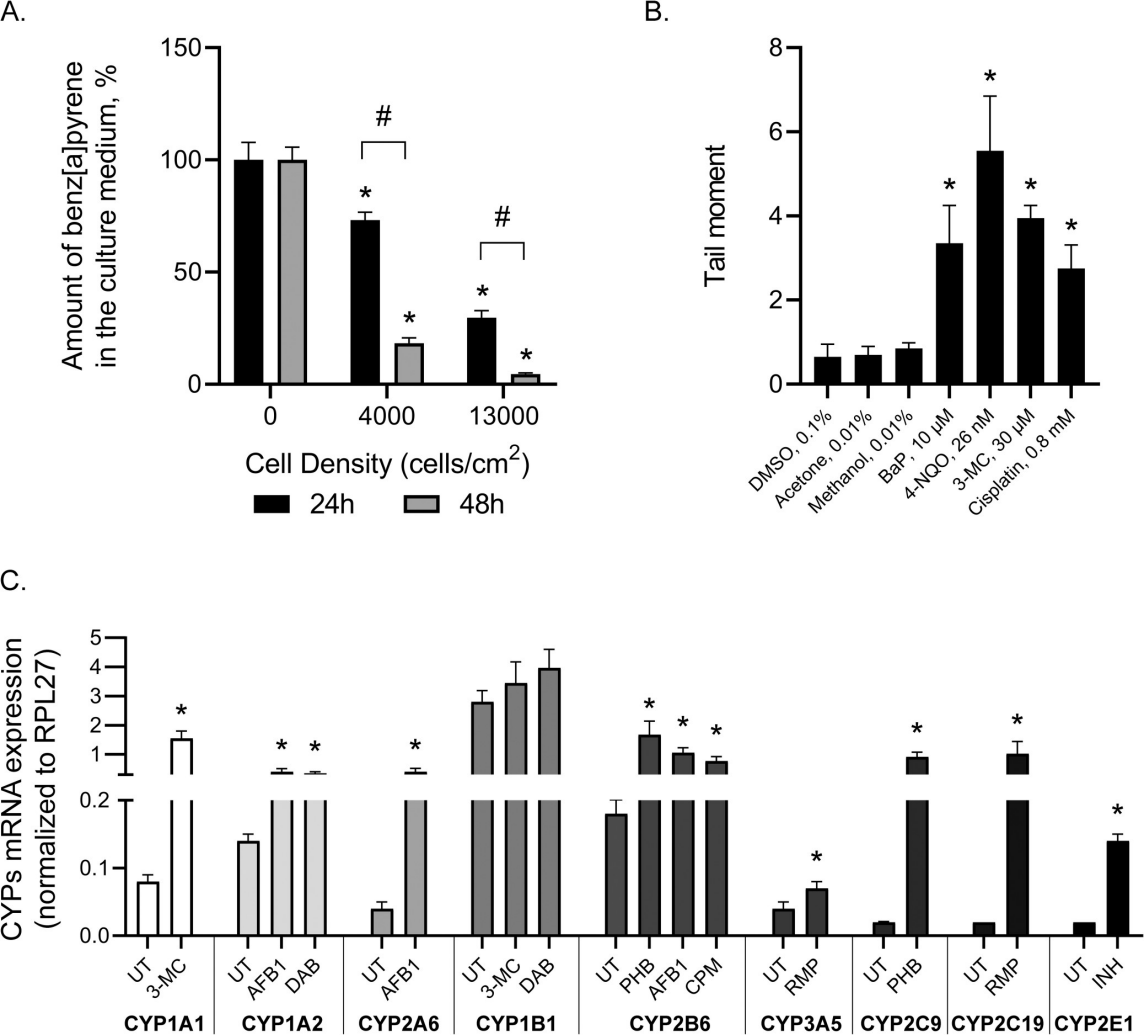
Metabolic competence of HeLa TI cells. A. Metabolism of procarcinogen benzo[a]pyrene by HeLa TI cells; Shpol’skii method. B. Level of DNA damage in HeLa TI cells after carcinogen and procarcinogen treatment; Comet assay. C. Activity of different CYP450 isoform genes in HeLa TI cells after treatment with microsomal oxygenase inductors, qRT-PCR (results are presented as the fold change (2 −ΔCT) in the level of the expression, which was normalized to that of the *RPL27* gene). The values are expressed as the means ± SD, and “*” indicates the level of significance (p <0.05).

When the initial cell count of HeLa TI cells was 4×10^4^ per well, after 24 and 48 hours of culture, the amount of residual B[a]P decreased by 27% and 82%, respectively (Fig 5A). The increase in the HeLa TI initial cell number, up to 12×10^4^ per well, led to more intensive B[a]P metabolism: the amount of residual B[a]P after 24 and 48 hours of culture was decreased by 70% and 90%, respectively (Fig 5A). Thus, the amount of residual B[a]P in the culture medium depended both on the cell number and on the duration of the culture, confirming that HeLa TI cells harbor biotransformation enzymes.

### Genotoxic effects of procarcinogens in HeLa TI cells

Genotoxic procarcinogens that require metabolic activation could induce intensive DNA damage in cultured cells only when the cells are metabolically competent [52–54]. Therefore, the genotoxic effect induced by procarcinogens in HeLa TI cells is considered to be an indirect confirmation of cell metabolic competence.

Using different cell lines, a previous study has shown that benzo[a]pyrene and 3-methylcholanrene exhibit genotoxic potential when they undertake extensive metabolism [53–55]. A comet assay has been successfully applied for the assessment of DNA damage in metabolically competent cells exposed to procarcinogens [52,53].

Using a DNA comet assay, we assessed the levels of DNA damage after exposure of HeLa TI cells to the procarcinogens benzo[a]pyrene, 3-methylcholanrene and 4-nitroquinoline-n-oxide. Cisplatin, a direct-acting agent that is widely used in cancer chemotherapy, was used as the positive control, as previously described [56,57]. The DNA damage index (tail moment) in the HeLa TI cells exposed to B[a]P, 4-NQO and 3-MC increased by 3.4-, 5.6-, and 4.0-fold, respectively, compared to the control level observed in the cells treated with solvent (Fig 5B). After the treatment of HeLa TI cells with cisplatin, the DNA damage index increased by 2.75 (Fig 5B). Thus, our results obtained by comet assay demonstrated the metabolic competence of the HeLa TI cells.

### Expression of different cytochrome P450 isoforms in HeLa TI cells

In the first step of xenobiotic metabolism in human cells, cytochrome P450 (CYP) mono-oxygenases play key roles. The individual isoforms of this enzyme family exhibit distinct substrate selectivity and differ in terms of regulation of expression [44,58]. We assessed the constitutive and inducible expression levels of several cytochrome P450 isoforms playing a prominent role in the metabolism of drugs and environmental chemicals in HeLa TI cells. Using quantitative real-time PCR, we analyzed the expression levels of *CYP1A1*, *CYP1A2*, *CYP1B1*, *CYP2A6*, *CYP2B6*, *CYP2C9*, *CYP2C19*, *CYP2E1*, and *CYP3A5*. The constitutive expression level was revealed for all these isoforms except *CYP2C9*, *CYP2C19*, and *CYP2E1* (Fig 5C). Specific induction of the individual isoforms was performed by the treatment of HeLa TI cells with the inductor compounds presented in Table 1.

**Table 1:** mRNA induction of CYP isoforms in HeLa TI cells.

№	CYP isoform	Inductor		Fold change
		Compound	Abbreviation	
1	CYP1A1	3-Methylcholanthrene, 2 μM	3-МС	18.5*
2	CYP1A2	Aflatoxin B1, 0.2 μM	AFB1	2.8*
		3’,3-Diaminobenzidine, 80 μM	DAB	2.5*
3	CYP2A6	Aflatoxin B1, 0.2 μM	AFB1	9.3*
4	CYP1B1	3-Methylcholanthrene, 2 μM	3-МС	1.2
		3’,3-Diaminobenzidine, 80 μM	DAB	1.4
5	CYP2B6	Phenobarbital, 1 mM	PHB	9.5*
		Aflatoxin B1, 0.2 μM	AFB1	6.1*
		Cyclophosphamide, 0.15 mM	CPM	4.4*
6	CYP2C9	Phenobarbital, 1 mM	PHB	>40*
7	CYP2C19	Rifampicin, 0.35 mM	RMP	>40*
8	CYP2E1	Isoniazid, 100 mM	INH	>7*
9	CYP3A5	Rifampicin, 0.35 mM	RMP	1.6

*- the difference is significant (p<0.05).

After induction, the expression of almost all the isoforms increased, including those not constitutively expressed. Thus, we demonstrated that HeLa TI cells possess active and inducible cytochrome P450 monooxygenases.

### Assessment of the effects of the S9 mixture used for procarcinogen metabolic activation on GFP reactivation in HeLa TI cells

**Fig 6:**
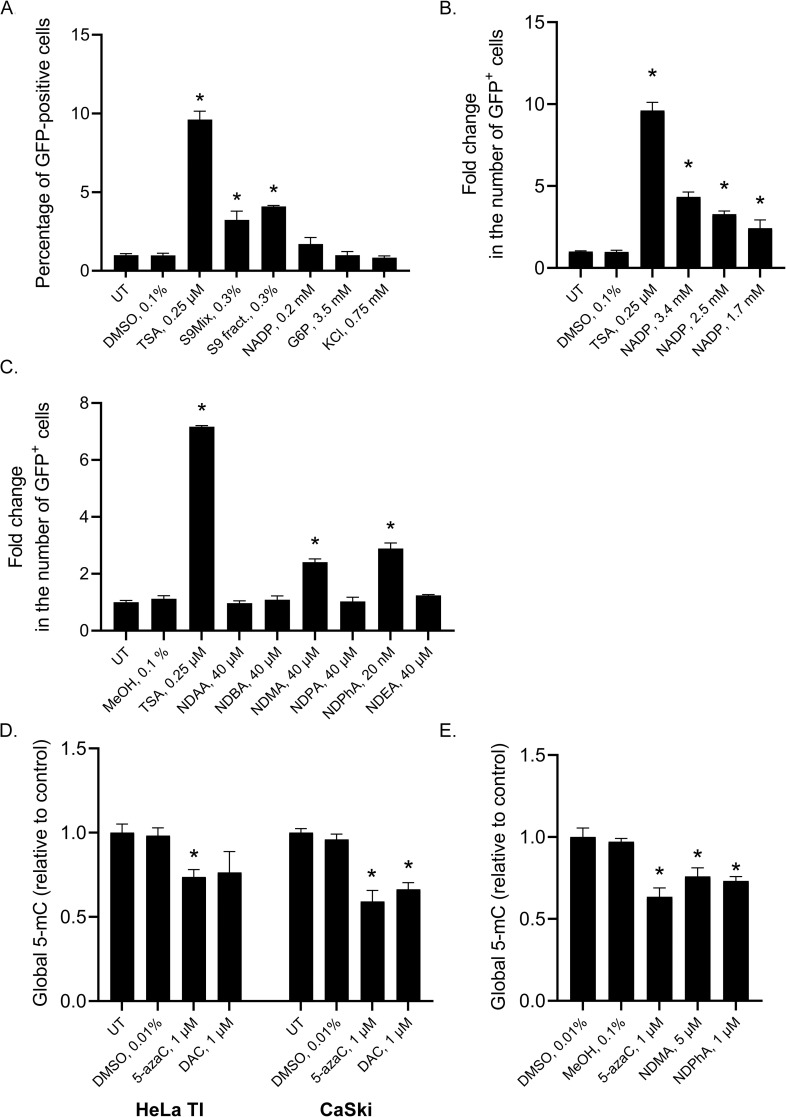
**A, B.** Effects of the S9 mixture and its components on *GFP* reactivation in HeLa TI cells. **C.** Effects of procarcinogens from the N-nitrosamine class on *GFP* reactivation in HeLa TI cells. GFP-positive cells were counted with flow cytometry. Data normalized to vehicle. **D.** Analysis of the sensitivity of HeLa TI and CaSki cells to the demethylating agents. **E.** Effects of nitrosamines NDMA and NDPhA on DNA methylation. The values are expressed as the means ± SD, and “*” indicates the level of significance (p <0.05).

It is recommended that in vitro systems for genotoxicity screening include the metabolic activation procedure that depends on the use of the rodent liver S9 fraction, which is a postmitochondrial supernatant consisting of both cytosol and microsomes [37,47,59]. The S9 fraction contains many chemical and enzyme components, among which the most important are NADP, glucose-6-phosphate and glucose-6-phosphate-dehydrogenase, required for the generation of NADPH, a cofactor for cytochrome P450. Taking into account that metabolic activation of xenobiotics should be considered in testing for epigenetic activity, we analyzed the influence of the rat liver S9 fraction and its NADPH-generating components on *GFP* expression reactivation in HeLa TI cells. TSA was used as a positive control. Our results showed a significant epigenetic effect of the S9 fraction: *GFP* expression was reactivated in 18% of HeLa TI cells (Fig 6A). Moreover, we observed a dose-dependent *GFP*-reactivating effect of NADP when it was added to the culture medium of the HeLa TI cells at nontoxic millimolar concentrations (Fig 6B). Thus, we found that the S9 fraction influences epigenetic silencing regulation and should therefore be specifically controlled when used in experiments.

### Assessment of the effect of procarcinogens on GFP reactivation in HeLa TI cells

For the analysis of the epigenetic activity of procarcinogens, we selected a group of nitrosamines from the class of Nitroso compounds. The list of investigated agents included probable carcinogens (IARC group 2A) N-nitrosodimethylamine (NDMA) and N-nitrosodiethylamine (NDEA), possible carcinogens (IARC group 2B) N-nitrosodiallylamine (NDAA), N- nitrosodibutylamine (NDBA), N-nitrosodipropylamine (NDPA) and a compound with unproven carcinogenicity (IARC group 3) N-nitrosodiphenylamine (NDPhA). Nitrosamine compounds are indirect-acting carcinogens that depend on metabolic activation, which makes them the most suitable substances for analyzing the ability of HeLa TI cells to reveal the epigenetic-modulating properties of procarcinogens. After treatment of the HeLa TI cells with NDMA and NDPhA, the number of GFP-positive cells increased by 2.4- and 3.0-fold, respectively (Fig 6C), which indicates the epigenetic modulating potential of these compounds.

### Assessment of the effect of nitrosamines that caused *GFP* gene reactivation on DNA methylation

A preliminary analysis of the sensitivity of HeLa TI cells to the demethylating agents 5-azacytidine and decitabine was carried out, and the results showed that these agents caused a decrease in the level of DNA methylation by 26% and 24%, respectively. According to literature data, the CaSki cell line is a cervical cancer cell line with less pronounced DNA hypomethylation [60]. An analysis of the sensitivity of CaSki cells to demethylating agents showed a statistically significant decrease in DNA methylation under the action of 5-azacytidine and decitabine, by 41% and 34%, respectively (Fig 6D). Thus, the study of the demethylating activity of nitrosamines that caused GFP gene reactivation was carried out on CaSki cells, as they are more sensitive to changes in the integral level of DNA methylation. The data obtained showed that under the action of nitrosamines NDMA and NDPhA, the degree of cytosine methylation in genomic DNA significant decreased, by 23% and 25%, respectively (Fig 6E).

## Discussion

The currently available test systems for the screening of epigenetically active compounds are mainly directed toward definitive epigenetic events or a limited number of events (site-specific DNA methylation, global DNA methylation, or definite modifications of histones). There is no test system that affords identification of chemicals influencing a wide range of different epigenetic enzymes, except for a test system developed by Martinez et al. [61], who performed simultaneous screening for small-molecule inhibitors of both HDACs and DNMTs. This test system was based on cell selection for vector insertion and *GFP* silencing, and it is highly likely that the number of epigenetic enzymes participating in *GFP* silencing of the stably transfected mouse C127 cells analyzed was not limited to the enzymes identified by the authors. In addition, the epigenetic enzymes involved in reporter gene silencing were not characterized, and mouse (not human) cells were used in the assay. To develop further cell-based reporter assays for epigenetic modulators, we used HeLa TI cells.

HeLa cells have been infected to obtain one integrated reported GFP gene per cell, and the number of cells was enriched after multiple cycles of sorting cells harboring epigenetically silenced GFP integrated in different cells at different sites [29]. Comparing different cell clones of the HeLa TI cell population, a previous study showed that silencing and repression can occur independently of the integration site or the promoter controlling the silent GFP reporter gene and that the location of integrated GFP can influence the degree of repressive effects as well as the ability to respond to reactivating stimuli [29,30]. GFP silencing/reactivation was also previously demonstrated to occur via CHAF1A, KMT1E, TIF1, RAD21, PBRM1, ZMYND8, MBD3, MBD1, RING1, KMT5C, TIF1, DNMT3A, KDM4A, HPH2, KDM2A, and HDAC treatment [31,32]. Considering this information, we proposed that the HeLa TI cell population is characterized by a high potential to reveal compounds that could reactivate epigenetically silent GFP via different mechanisms. This characterization is a great advantage of the proposed test system as a screening system. Comparing the HeLa TI test system with many other in vitro systems developed to reveal compounds influencing gene silencing by only one known mechanism, we would like to point out that hundreds of chromatin regulators (including epigenetic factors and chromatin remodeling enzymes) and transcription activators are currently considered to influence epigenetic silencing. Thus, many systems directed at revealing only one mechanism of chemical action should be used within a battery of test systems to screen new chemicals for possible influence on epigenetic silencing. It makes screening time-consuming and expensive. We propose to separate the screening procedure into two stages. In the first stage, a wide spectrum of chemicals and chemical mixtures for silent gene reactivation are to be screened qualitatively (+/-) using the HeLa TI system (or a similar broadband system), and in the second stage, the chemicals that reactivate gene expression in the qualitative test, are to be analyzed to elucidate the peculiar mechanism and to evaluate the effect using different test systems for specific activity. This two-step process may be used for a directed search of beneficial activity of new epigenetic drugs or for a toxicological study for the elucidation of hazardous activity. It will make the whole procedure of screening for epigenetic activity faster and less expensive.

The HeLa TI system is based on retroviral infection. During the last decade, mechanistic insights into epigenetic regulation of genome function have changed, and currently endogenous retroviral elements and highly repeated short and long interspersed sequences are considered important genome components regulated epigenetically to influence the cell transcriptome and proteome. Exposure to epigenetically active agents has been shown to produce genome-wide transcriptional and epigenomic consequences. Studies have revealed that more than 2000 transcription start sites not annotated previously were found both in cultured cancer cells treated with DNMTis and HDACis and in peripheral blood mononuclear cells (PBMNCs) of cancer patients treated with the HDACi vorinostat [62,63]. In particular, activation of human endogenous retroviral elements (HERVs) with concomitant synthesis of double-stranded RNAs and massive activation of promoters from long terminal repeats (LTRs) were induced by epigenetic modulators [63]. More than 3000 HERV sequences and 650000 LTRs have been identified in the human genome, and 5–8% of the genome sequences are similar to those of infectious retroviruses [63–65]. To maintain genome integrity, transposition of retroviral elements is prevented by epigenetic surveillance mechanisms, including DNA methylation and repressive histone modifications. In addition, HERVs are not universally silenced. In particular, their expression may be observed during embryonic development. It is also believed that the expression of HERVs is associated with a number of diseases [64,66,67]. Thus, epigenetic silencing of HERVs and LTRs is essential for genome function. HERV and retrotransposon silencing by packaging into heterochromatin has been described as requiring enzymes considered to be the main components of the epigenetic regulation system [62,63]. In our study, we demonstrated *GFP* reactivating effects for the majority of known inhibitors of these enzymes. Moreover, we demonstrated the effects of UNC0638, BIX01294, and DZNep, correspondingly inhibiting GLC, G9a, and EZH2, which are critical for the histone modifications characteristic of transcription repression, and revealed *GFP* reactivation by JQ-1, which is a bromodomain and extraterminal motif inhibitor. It seems that strong reactivation of the GFP gene after the treatment of cells with the agents JQ-1 and JQ-35 is not associated with the functional activity of BETis, which are primarily aimed at repressing transcription coactivator enzymes. Nevertheless, Banerjee et al showed that JQ-1 activates the transcription of a cluster of genes critical for chromatin organization. Moreover, enzymes encoded by activated genes do not belong to any particular class in terms of functionality and exhibit additional multidirectional epigenetic effects [68]. However, these data, together with the effects shown using the HeLa TI test system, demonstrate that BET inhibitors (we assume similar mechanisms of action for JQ-1 and JQ-35) are able to integrally influence chromatin organization and, as a consequence, profile gene expression.

However, 3D chromatin organization is an important physical driver determining the distribution of integration sites, and proviral integration sites are localized predominantly in euchromatin [69]. This may explain why we did not observe any effects of A-196, a known inhibitor of SUV420H1/2, which are critical for trimethylation of lysine 20 of H4 (H4K20me3), the mark of heterochromatin.

The overall ability of HeLa TI cells to reveal the effects of inhibitors of many different epigenetic enzymes represents a great advantage of this test system compared to other in vitro systems. The HeLa TI cell population represents a cell-based reporter test system for efficient, rapid and inexpensive screening of the epigenetic activity of xenobiotics. This system has been successfully applied for the study of the epigenetic effect of the new perspective chemotherapeutic drug curaxin CBL0137 [70] and of a number of minor groove-binding ligands [71].

We assessed the dose- and time-dependence of the TSA reactivating effect on HeLa TI cells to estimate the competence/sensitivity of the assay for the screening of epigenetic modulators [35]. We observed a linear dose dependence of the reactivating effect of TSA applied at a wide concentration range. We also observed the time dependence of *GFP* reactivation: the optimal TSA treatment duration was 24–72 hours, as during the first hours, *GFP* transcription was activated, while the cells synthesized and accumulated the protein. TSA has been shown to affect histone modifications near the gene promoter and increase the overall histone acetylation level in cells but does not influence DNA methylation levels [72].

One of the advantages of the proposed cell-based test system is metabolic competence. Only a small group of exogenous chemicals is excreted unchanged in urine or feces without any metabolic degradation, and the majority of these undergo biotransformation to active metabolites that ultimately to lead to biological effects [33]. It was established that the majority of carcinogens are chemically stable and require bioactivation to be capable of interacting with DNA [34]. We demonstrated HeLa TI metabolic competence both through an analysis of the concentration decrease of the parent procarcinogen B[a]P in the culture medium and by showing the genotoxic effect of procarcinogens B[a]P and 4-NQO, which could be induced with only activated metabolites. Moreover, we analyzed the constitutive and induced expression of nine cytochrome P450 isoforms in HeLa TI cells, which are most important for xenobiotic metabolism. Our results concerning CYP1A1 and CYP1B1 constitutive expression in HeLa TI cells are concordant with previously published data showing constitutive expression of these isoforms in HeLa cells [43,44], although we found more intensive induction of CYP1A1 by 3-MC (18.5-fold) compared to the results of Iwanari et al. (1.5-fold) [44]. Moreover, in contrast to the data of Iwanari et al., we demonstrated constitutive expression of CYP1A2, which was induced by aflatoxin B1 at 2.8-fold greater levels and by 3’,3-diaminobenzidine at 2.5-fold greater levels in our study. Constitutive and induced expression levels of the other CYP isoforms in HeLa TI cells were described in our study for the first time. Overall, the data obtained by all three approaches for assessing cell metabolic activity showed that HeLa TI cells are metabolically competent.

Our results concerning the activity of the S9 rat liver fraction in the HeLa TI test system were unexpected, as this fraction is widely used in genotoxic assays, and its epigenetic effect has not been described before. Moreover, NADP was epigenetically active in a dose-dependent manner. These results mean that usage of the S9 fraction and NADP in the assays for epigenetic activity of xenobiotics should be carefully controlled.

The usefulness of the HeLa TI system is demonstrated by its application in practice. We screened the epigenetic activity of 6 compounds from the nitrosamine group. Nitrosamines are well-known environmental pollutants with potentially vast coverage, with reports of their detection in tobacco products, food, water, etc. The formation of active carcinogenic molecules from nitrosamines occurs by the action of CYP2B, CYP2E1, CYP2A6, etc. [73]. We showed, for the first time, that N-nitrosodimethylamine and N-nitrosodiphenylamine affect epigenetic silencing. Based on the data obtained, it can be assumed that the carcinogenic properties of NDMA may be due not only to genotoxic but also to epigenetic effects, which can significantly expand the understanding of the concept of “epigenetic carcinogens”. Despite lack of evidence showing the carcinogenicity of NDPhA in humans in the literature, this agent is of significant interest for research. NDPhA has been found to be a potent transnitrosating agent resulting in a multifold increase in the effects of other nitrosamines [74]. On this basis, the epigenetic activity shown for NDPhA in the HeLa TI test system provides new insight into the mechanisms of action of this agent. As tobacco-specific nitrosamines are known to cause integral DNA demethylation [75], we analyzed the effect of NDPhA and NDMA on DNA methylation status and found a significant decrease in the degree of cytosine methylation of genomic DNA.

Discussing the limitations of the proposed test system, we need to point out that the origin of HeLa cells determined our system characteristics. It would be interesting to compare characteristics of TI populations obtained using cultured cell lines of different histology. In particular, HeLa TI cells are hypomethylated; thus, we recommend using cells with hypermethylated status for detailed analysis of xenobiotic effects on DNA methylation.

From the perspective of application of the HeLa TI test system, we propose its use both for wide screening of epigenetically active xenobiotics and, after the active compounds are revealed, further studying the dose-dependent changes in the epigenetic pattern and alterations in the main cell signaling pathways in the HeLa TI cells.

## Supporting information

S1 Fig (TIF)

S1 Table Primers sequences. (DOCX)

S2 Table Mechanisms of action of analyzed epigenic modulators. (DOCX)

S3 Table IC10 of analyzed epigenetic modulators. (DOCX)
