# Supplementary figures and images for: HeLa TI cell-based assay as a new approach to screen for chemicals able to reactivate the expression of epigenetically silenced genes
Source: PLoS One. 2021 Jun 11;16(6):e0252504. doi: 10.1371/journal.pone.0252504 (PMC8195432; doi:10.1371/journal.pone.0252504)

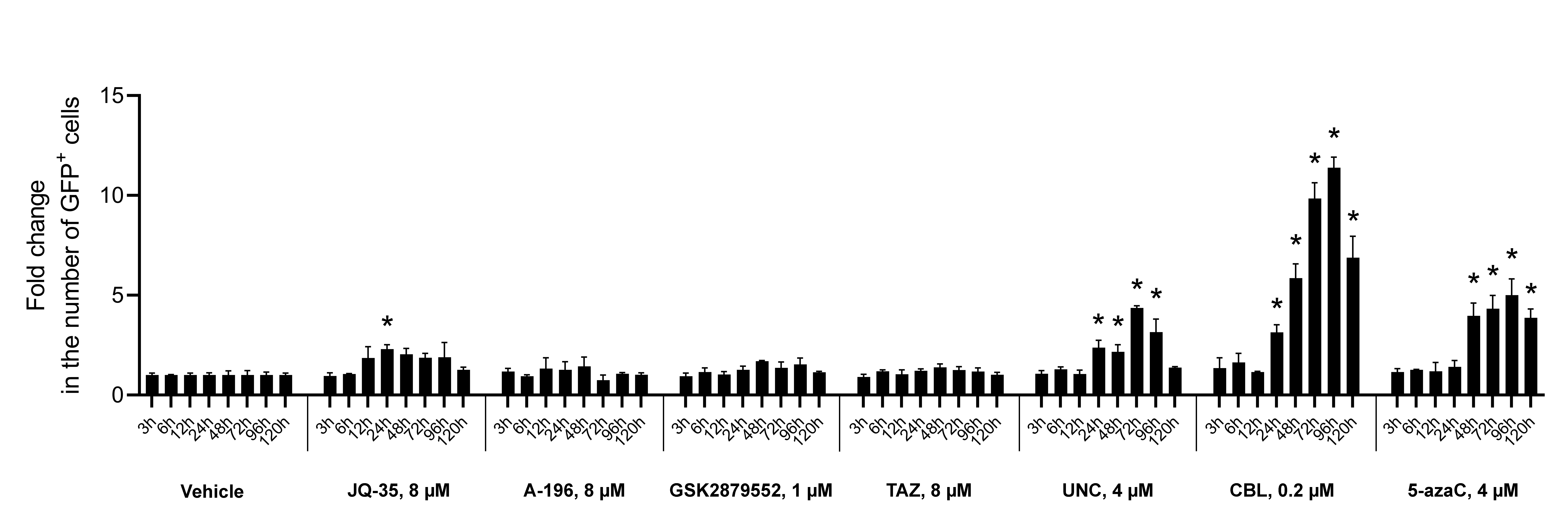

Supplement: S1 Fig — (TIF) [file pone.0252504.s001.tif]
