# Supplementary material for: HeLa TI cell-based assay as a new approach to screen for chemicals able to reactivate the expression of epigenetically silenced genes
Source: PLoS One. 2021 Jun 11;16(6):e0252504. doi: 10.1371/journal.pone.0252504 (PMC8195432; doi:10.1371/journal.pone.0252504)
Supplement: S1 Table — (DOCX) [file pone.0252504.s002.docx]

**Table S1. Primers sequences.**

| Gene | Forward sequence (5’-3’) | Reverse sequence (5’-3’) | NCBI gene ID |
| --- | --- | --- | --- |
| *RPL27* | ACCGCTACCCCCGCAAAGTG | CCCGTCGGGCCTTGCGTTTA | NM_001349921.2 |
| *CYP1A1* | TCCAGAGACAACAGGTAAAACA | AGGAAGGGCAGAGGAATGTGAT | NM_000499.5 |
| *CYP1A2* | AGTCCAGGAGCACTATCAGG | AAGGTACATGAGGCTCCAGG | NM_000761.5 |
| *CYP1В1* | AACGTACCGGCCACTATCAC | TCACCCATACAAGGCAGACG | NM_000104.4 |
| *CYP2A6* | CCTCATGAAGATCAGTGAGCGCTAT | GCTCCCCGTTGCTGAATACC | NM_000762.6 |
| *CYP2B6* | ATGCCAATGGGGCACTGAA | ATTTTGCCCACACCACACTC | NM_000767.5 |
| *CYP2С9* | CTTGGAAAACACTGCAGTTGAC | CACAGCATCTGTGTAGGGCA | NM_000771.4 |
| *CYP2С19* | CATCAACAACCCTCGGGACTT | GTCAGCTGCAGTGATTACCAAG | NM_000769.4 |
| *CYP2Е1* | AAGCGCTGCTGGACTACAAG | GGAGGGTGGTCAGGGAAAAC | NM_000773.4 |
| *CYP3А5* | CTCCTCTATCTATATGGGACCCG | CAGCACAGGGAGTTGACCTT | NM_001190484.3 |
