# Supplementary material for: HeLa TI cell-based assay as a new approach to screen for chemicals able to reactivate the expression of epigenetically silenced genes
Source: PLoS One. 2021 Jun 11;16(6):e0252504. doi: 10.1371/journal.pone.0252504 (PMC8195432; doi:10.1371/journal.pone.0252504)
Supplement: S2 Table — (DOCX) [file pone.0252504.s003.docx]

**Table S2. Mechanisms of action of analyzed epigenic modulators.**

| Function | Group | **Agent** | Target enzyme | Modification **realized by the target enzyme** | **Modification** function | **Agent** function |
| --- | --- | --- | --- | --- | --- | --- |
| Writer | Histone methyltransferases inhibitors (HMTis) | UNC-0638 | G9a, GLC | H3K9me2 | Repression of transcription in euchromatin | Inhibition of the enzyme that promotes the repression of transcription |
|  |  | BIX-0124 | G9a | H3K9me2 | Repression of transcription in euchromatin | Inhibition of the enzyme that promotes the repression of transcription |
|  |  | Tazemetostat | EZH2 | H3K27me3 | Repression of transcription in heterochromatin | Inhibition of the enzyme that promotes the repression of transcription |
|  |  | DZNep | EZH2 | H3K27me3, H4K20me3 | Repression of transcription in heterochromatin | Inhibition of the enzyme that promotes the repression of transcription |
| Reader | Bromodomains and Extra-Terminal motif inhibitors (BETis) | JQ-35 | BET | H3ac | BET family proteins are transcriptional coactivators | Inhibition of transcription coactivator enzyme |
|  |  | JQ-1 | BET | H3ac | BET family proteins are transcriptional coactivators | Inhibition of transcription coactivator enzyme |
| Writer | Histone methyltransferases inhibitors (HMTis) | A-196 | SUV420H1/2 / KMT5B | H4meK20me3 | Repression of transcription in heterochromatin | Inhibition of the enzyme that promotes the repression of transcription |
| Eraser | (Lysine demethylases inhibitor) KDMi | GSK2879552 | KDM1A | Demethylation H3K4me1/2, Demethylation H3K9me1/2 | H3K4me1, me2; H3K9me1 corresponds to actively transcribed regions of euchromatin, H3K9me2 corresponds to heterochromatin. Demethylation of these marks has the opposite meaning | Mixed effect |
| Eraser | Histone deacetylases inhibitors (HDACis) | Sirtinol | SIRT 1, SIRT 2 | Deacetylation H3, Deacetylation H4 | H3ac - associated with euchromatin, => deacetylation of H3 leads to repression of transcription in euchromatin | Inhibition of the enzyme that promotes the repression of transcription |
|  |  | Sodium butyrate | HDAC1, HDAC2, HDAC3 |  |  |  |
|  |  | Depsipeptide | HDAC1, HDAC2 |  |  |  |
|  |  | Entinostat | HDAC1, HDAC3 |  |  |  |
|  |  | Pomiferin | HDACs |  |  |  |
|  |  | Vorinostat | HDAC1, HDAC2, HDAC3, HDAC6, HDAC8, HDAC 10, HDAC11 |  |  |  |
|  |  | Trichostatin A (TSA) | HDAC1, HDAC2, HDAC3, HDAC4, HDAC6, HDAC7, HDAC9, HDAC10 |  |  |  |
|  |  | Valproic acid (VPA) | HDAC1, HDAC2, HDAC3, HDAC4, HDAC5, HDAC7, HDAC8, HDAC9 |  |  |  |
| Writer | DNA-methyltransferase inhibitors (DNMTis) | 5-Azacytidine | DNMTs | DNA methylation | Repression of transcription | Inhibition of the enzyme that promotes the repression of transcription |
|  |  | Decitabine (DAC) | DNMTs | DNA methylation | Repression of transcription |  |
|  |  | RG108 | DNMTs | DNA methylation | Repression of transcription |  |
| - | Chromatin remodeler | Curaxin CBL0137 | histone chaperone facilitates chromatin transcription (FACT) | - | - | Inhibition of FACT and nucleosome relaxation |
