# Supplementary material for: HeLa TI cell-based assay as a new approach to screen for chemicals able to reactivate the expression of epigenetically silenced genes
Source: PLoS One. 2021 Jun 11;16(6):e0252504. doi: 10.1371/journal.pone.0252504 (PMC8195432; doi:10.1371/journal.pone.0252504)
Supplement: S3 Table — (DOCX) [file pone.0252504.s004.docx]

**Table S3. IC10 of analyzed epigenetic modulators**

| Group | Compound | IC10, μM |
| --- | --- | --- |
| DNA-methyltransferase inhibitors (DNMTis) | 5-Azacytidine | 5 |
|  | DAC (Decitabine) | 5 |
|  | RG108 | 5 |
| Histone deacetylases inhibitors (HDACis) | Depsipeptide | 5 |
|  | Entinostat | 5 |
|  | Pomiferin | 5 |
|  | Sodium butyrate (NaB) | 5000 |
|  | Sirtinol | 4 |
|  | Trichostatin A (TSA) | 0,2 |
|  | Valproic acid (VPA) | 5 |
|  | Vorinostat | 5 |
| Histone methyltransferases inhibitors (HMTis) | A-196 | 8 |
|  | BIX-0124 |  |
|  | DZNep | 5 |
|  | Tazemetostat | 8 |
|  | UNC-0638 | 4 |
| (Lysine demethylase inhibitor) KDMi | GSK2879552 | 8 |
| Bromodomains and Extra-Terminal motif inhibitors (BETis) | JQ-35 | 8 |
|  | JQ-1 | 5 |
| Chromatin remodeler | Curaxin CBL0137 | 0,2 |
